# Cutaneous inflammation alters nociceptor electrophysiology in guinea pigs but not rats

**DOI:** 10.17305/bb.2025.12195

**Published:** 2025-05-19

**Authors:** Laiche Djouhri, Ahmed Eliwa, Alghalya Al-Emadi, Yehia Y Hussein, Hissa Al-Suwaidi, Al-Jouhara Albaloshi, Ayman Mustafa, Mohammed Seed Ahmed

**Affiliations:** 1 Department of Basic Medical Sciences, College of Medicine, QU Health, Qatar University, Doha, Qatar; 2 College of Medicine, QU Health, Qatar University, Doha, Qatar; 3 Department of Orthopedic Surgery, Hamad Medical Corporation, Doha, Qatar

**Keywords:** Tissue inflammation, nociception, inflammatory pain, *in vivo* electrophysiology, nociceptors

## Abstract

Inflammatory pain hypersensitivity is believed to result, in part, from increased excitability of nociceptive dorsal root ganglion (DRG) neurons. We previously demonstrated in guinea pigs that hindlimb inflammation induces electrophysiological changes in these neurons, including faster action potential (AP) and afterhyperpolarization (AHP) kinetics. Given that rats and guinea pigs are distinct species with notable differences in genetic composition and physiology, we hypothesized that cutaneous inflammation would have different effects on the electrophysiological properties of nociceptive DRG neurons in rats—the predominant rodent model for pain research. To test this hypothesis, we performed intracellular voltage recordings from DRG neurons (*n* ═ 430) in deeply anesthetized, untreated (control) and complete Freund’s adjuvant (CFA)-treated rats and guinea pigs. C-, Aδ-, and Aβ-nociceptors were identified based on their dorsal root conduction velocities (CVs) and responses to natural noxious stimuli. Consistent with our hypothesis, we observed no significant changes in any electrophysiological variables in rat nociceptive neurons four days after CFA-induced hindlimb inflammation. In contrast, guinea pig nociceptors exhibited a significant increase in CV and significant decreases in both AP and AHP durations. The inflammation-induced shortening of absolute and relative refractory periods likely contributes to increased firing frequency in nociceptive nerve fibers, thereby promoting inflammatory pain hypersensitivity. These findings suggest species-specific differences in peripheral neuronal mechanisms underlying inflammatory pain, potentially due to variation in ion channel expression and/or function in DRG neurons between rats and guinea pigs. Given the genetic and metabolic similarities between guinea pigs and humans, further research is warranted to determine whether guinea pigs may serve as a more accurate model of chronic inflammatory pain than rats.

## Introduction

Chronic or persistent inflammatory pain may result from peripheral tissue injury or inflammation. A key feature of this condition is the presence of spontaneous (ongoing) pain and hypersensitivity to stimuli that are normally nonpainful (allodynia) or painful (hyperalgesia) [1, 2]. Although the underlying neuronal mechanisms of chronic inflammatory pain are not fully understood, preclinical studies using animal models suggest that it partly results from phenotypic changes at multiple levels of the nociceptive pathway. These changes include increased excitability of nociceptive primary afferent neurons (peripheral sensitization) and central neurons (central sensitization) [1, 3–5]. Notably, central sensitization is believed to be partly driven by input from spontaneously active C-fiber afferents [5–7]. During chronic inflammation, primary afferent neurons become hyperexcitable and begin generating spontaneous activity (SA)—abnormal, spontaneous nerve impulses or action potentials (APs)—which is a hallmark of neuronal hyperexcitability. Using the complete Freund’s adjuvant (CFA) model of inflammatory pain, we and others have shown that both C- and A-fiber dorsal root ganglion (DRG) neurons exhibit SA following persistent cutaneous inflammation [8–11]. Moreover, we previously demonstrated that SA in rat C-nociceptors correlates with spontaneous pain behavior in the CFA model of inflammatory pain [9]. Additional changes in the somata and fibers of DRG neurons innervating inflamed tissue have also been reported and are believed to contribute to chronic inflammatory pain. These include changes in the chemical phenotype of rat Aβ-fibers [12] and alterations in the electrophysiological membrane properties of nociceptive DRG neurons in the guinea pig [8, 13]. The electrophysiological changes we previously reported in guinea pig nociceptors include faster AP and afterhyperpolarization (AHP) kinetics, as well as increased conduction velocities (CVs) [8, 13]. As we have suggested, these changes likely enhance the ability of nociceptors to transmit information to the CNS, thereby contributing to inflammatory pain [8, 13]. Rats and guinea pigs are genetically, biologically, and behaviorally distinct species [14]. For example, rats are social animals known for their agility, climbing skills, and complex social interactions [15]. In contrast, guinea pigs are more passive, less agile, and exhibit a more sedentary disposition [16]. Although guinea pigs are, in several respects—such as genetics and metabolism—more similar to humans than rats, mice, and even chimpanzees [17], rats and mice remain the most commonly used rodent models in biomedical pain research (see, e.g., [18]). Given that rats are the predominant rodent model for pain research and that guinea pigs are phylogenetically distinct [19, 20], the aim of this study was to determine whether the electrophysiological changes observed in guinea pig DRG nociceptors following CFA-induced inflammation also occur in rats. To this end, we conducted intracellular recordings from the somata of lumbar C-, Aδ-, and Aβ-fiber DRG neurons in deeply anesthetized normal and CFA-treated rats, and compared their electrophysiological properties to those observed in guinea pig nociceptors four days post-CFA.

## Materials and methods

### Animals

*In vivo* electrophysiological experiments were conducted on female Wistar rats (180–300 g, Charles River, UK) at the University of Liverpool and female Dunkin-Hartley guinea pigs The animals were housed in cages with soft bedding and had ad libitum access to food and water. Room temperature was maintained between 20 ^∘^C and 26 ^∘^C, under a 12-h light/dark cycle. All experimental protocols were approved by the respective ethical review committees of the University of Liverpool and the University of Bristol, and complied fully with the UK Home Office Guidelines and the Animals (Scientific Procedures) Act 1986.

### Animal model of chronic inflammatory pain

We used the CFA model, which involved two intradermal injections of CFA (Sigma, St. Louis, MO, USA) administered under anesthesia with 4% halothane. Injections were delivered within the cutaneous receptive fields of the L4 and L5 DRGs in rats, and the L6 and S1 DRGs in guinea pigs. The first injection (100 µL) was made into the plantar surface of the left hindpaw, and the second (100 µL) into the left knee region. This procedure was designed to induce unilateral inflammation throughout the left hindlimb, as previously described [11, 13]. CFA was prepared as a 0.5 mg/mL suspension in an oil/saline (1:1) emulsion. Each milliliter of CFA solution contained 1 mg of heat-killed and dried Mycobacterium tuberculosis, 0.15 mL of mannide monooleate, and 0.85 mL of paraffin oil. Control animals did not receive CFA treatment. Notably, the two intradermal CFA injections produced a localized area of erythema and edema, with a mean 20% increase in girth of the ipsilateral foot compared to the contralateral foot [13]. These inflammatory symptoms were not observed in the hip. Following CFA injection, animals were prepared for *in vivo* electrophysiological recordings as described below.

### *In vivo* electrophysiology

Full details of the surgical procedures and animal preparation for *in vivo* electrophysiological recordings from DRG neurons were as previously described for the rat (e.g., [11]) and guinea pig [13, 21]. Briefly, animals were initially anesthetized with sodium pentobarbitone (60 mg/kg, i.p.) and maintained under deep anesthesia throughout the experiments with supplementary doses (10 mg/kg, i.a.) administered hourly. Deep anesthesia was confirmed by the complete absence of limb withdrawal reflex (areflexia). As the initial dose of anesthetic depresses ventilation, a tracheotomy was performed immediately following induction to allow artificial ventilation and continuous monitoring of end-tidal CO_2_. The left jugular vein and carotid artery were cannulated for intravenous administration of additional anesthetic and for blood pressure monitoring, respectively. During electrophysiological recordings, animals were paralyzed using either pancuronium (0.5 mg/kg, i.a.) or gallamine triethiodide (Flaxedil; 2 mg/kg, i.a.). Muscle relaxants were always administered alongside an additional dose of anesthetic (10 mg/kg, i.a.) every hour. The same dosage and timing of supplementary anesthetic were used both before and during paralysis, maintaining consistent areflexia. Core body temperature was maintained at 36 ± 0.5 ^∘^C. DRG Exposure and Recording: The procedure for exposing and stabilizing the DRGs was as previously described [13]. Briefly, following a laminectomy, the dorsal root of the DRG under investigation was cut close to its entry into the spinal cord and laid across a pair of stimulating platinum electrodes. The exposed nervous tissue (DRGs, dorsal root, and spinal cord) was protected using liquid paraffin within a paraffin pool formed with dental impression material. Recordings were performed four days after CFA treatment in rats and guinea pigs, and in age- and weight-matched untreated animals. Intracellular voltage recordings of somatic APs were made using sharp glass micropipettes filled with 1 M KCl (electrode resistance: 50–120 MΩ). Somatic APs were evoked antidromically by stimulating the dorsal root with single rectangular pulses: 0.03 ms for A-fiber units and 0.3 ms for C-fiber units. Stimulus intensities were set to twice the threshold for A-fibers and 1.5 times threshold for C-fibers. Neurons exhibiting high-frequency injury discharge were excluded from analysis. The temperature of the paraffin pool near the recorded DRG was maintained between 30 ^∘^C and 32 ^∘^C. APs were recorded in real-time using a Cambridge Electronic Design (CED, Cambridge, UK) 1401plus interface, and subsequently analyzed offline using the CED Spike2 software, as described previously [11, 13].

### Electrophysiological variables measured

**Figure 1.**
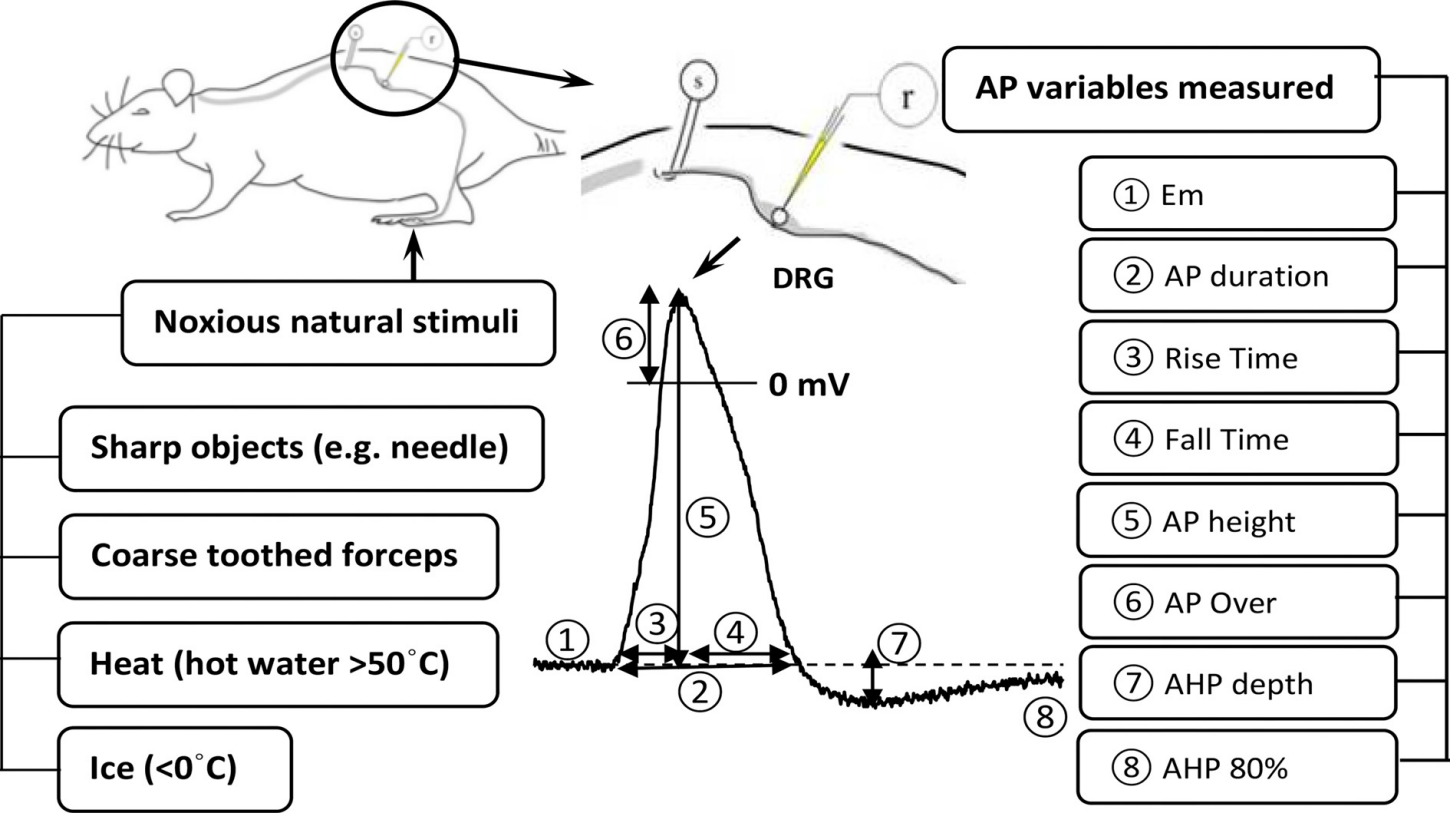
**A diagram showing the *in vivo* intracellular recording setup.** A glass microelectrode is inserted into a lumbar DRG neuron for intracellular recording (r) of somatic APs evoked antidromically by electrical stimulation of the dorsal root with a pair of bipolar platinum stimulating electrodes (s). The numbers on the intracellularly recorded somatic AP (middle) show the AP variables measured: (1) membrane potential (Em), (2) AP duration at base, (3) AP rise time, (4) AP fall time, (5) AP height/amplitude, (6) AP overshoot, (7) AHP depth and (8) AHP 80% (AHP duration to 80% recovery). The diagram also shows (left) the various noxious mechanical and thermal stimuli that were applied to the left hindlimb to classify neurons into different subtypes of nociceptors. AP: Action potential; DRG: Dorsal root ganglion; AHP: Afterhyperpolarization.

A number of electrophysiological variables were measured, including the following: (1) membrane potential (Em), (2) AP duration at the base (APdB), (3) AP rise time (RT), (4) AP fall time (FT), (5) AP height/amplitude, (6) AP overshoot, (7) AHP depth/amplitude, and (8) AHP duration to 80% recovery (AHP80%) (see Figure 1 and Table 1). In addition, the CV of each neuron was calculated by dividing the conduction distance—measured at the end of each experiment as the distance from the stimulating electrode to the recording site in the DRG, typically 4–7 mm—by the latency between the stimulus artifact and the onset of the evoked AP.

**Table 1:** Impact of CFA-induced hindlimb inflammation on electrophysiological properties of nociceptive DRG neurons in the guinea pig and rat

**Animal/ CV group**	**Animal** **group**	**N**	**CV** **m/s**	**Em** **- (mV)**	**AP height** **(mV)**	**AP overshoot** **(mV)**	**AP duration** **at base (ms)**	**Rise time** **(ms)**	**Fall time** **(ms)**	**AHP depth** **(mV)**	**AHP duration 80% (ms)**
Rat C-fiber	Normal	34	0.4 (0.4–0.6)	54 (61–48)	76 (69–83)	23 (11–28)	5.3 (3.9–6.9)	1.9 (1.4–2.6)	3.3 (2.2–4.7)	7 (3.5–9)	17 (10–28)
	CFA	43	0.4 (0.4–0.5)	52 (61–47)	79 (71–91)	27 (20–35)	5.6 (4.0–7.6)	2.0 (1.5–2.7)	3.4 (2.4–4.6)	7 (4.8–11)	24 (15–31)
Guinea pig C-fiber	Normal	41	0.4 (0.3–0.5)	46 (51–42)	67 (60–73)	19 (14–26)	5.0 (3.7–6.1)	2.1 (1.7–2.8)	2.8 (2.0–3.3)	8 (5.1–12)	18 (13–28)
	CFA	23	0.4 (0.3–0.7) *	48 (52–42)	72 (62–80)	21 (18–33)	3.7 (3.3–5.0) **	1.8 (1.6–2.1) *	1.9 (1.7–2.9) **	10 (8.1–12	12 (10–20) *
Rat Aδ-fiber	Normal	58	5.0 (4–6)	45 (55–45)	68 (59–81)	19 (9–30)	2.6 (2.1–3.2)	1.0 (0.9–1.4)	1.5 (1.2–1.9)	9 (6.5–10)	16 (7–61)
	CFA	24	5.0 (4–6)	54 (62–47)	77 (69–85)	21 (13–23)	2.6 (1.9–3.1)	1.1 (0.8–1.9)	1.5 (1.2–1.9)	8 (4.8–11)	26 (10–55)
Guinea pig Aδ-fiber	Normal	50	3.0 (2–4)	49 (53–44)	72 (63–77)	20 (15–26)	2.8 (2.3–3.3)	1.2 (0.9–1.4)	1.5 (1.3–1.9)	9 (4.7–12)	14 (10–22)
	CFA	19	2.9 (2–4)	50 (56–42)	68 (54-75)	25 (11–30)	2.2 (1.7–2.4) **	0.9 (0.6–1.1) *	1.2 (1.1–1.4) **	8 (6.0–12)	11 (6.0–15) *
Rat Aα/β-fiber	Normal	40	11 (8–15)	56 (60–49)	71 (61–82)	16 (9.0–24)	1.6 (1.3–2.2)	0.7 (0.5–1.0)	0.9 (0.8–1.1)	9 (7.1–10)	15 (8.0–37)
	CFA	26	10 (8–15)	54 (63–49)	72 (64–80)	15 (5.0–28)	1.5 (1.3–2.0)	0.6 (0.5–0.9)	0.8 (0.7–1.1)	8 (6.5–12)	11 (5.0–30)
Guinea pig Aα/β-fiber	Normal	51	6.0 (4.9–8)	49 (55–44)	72 (61–76)	20 (11–26)	2.02 (1.5–2.4)	0.8 (0.6–1.1)	1.2 (0.9–1.5)	8 (6.6–11)	13 (8.5–22)
	CFA	21	7.0 (6.0–9)	47 (53–44)	60 (53–75)	15 (7–27)	1.7 (1.3–2.0) *	0.7 (0.5–0.9) *	1.0 (0.8–1.3) **	7 (4.6–8)	10 (6.3–16)

CFA: Complete Freund’s adjuvant; DRG: Dorsal root ganglion; AP: Action potential; AHP: Afterhyperpolarization.

### Sensory receptive properties of DRG neurons

The sensory receptive properties of DRG neurons were examined using hand-held stimulators and classified as previously described in the guinea pig [13] and rat [22]. Natural noxious mechanical and thermal stimuli were applied to identify nociceptive neurons. These stimuli included pinching with fine or coarse-toothed forceps, sharp objects (e.g., a needle), noxious heat (hot water at 50 ^∘^C or a heated glass rod), and noxious cold (<0 ^∘^C). Nociceptive neurons were categorized into two main groups: (1) Aβ-, Aδ-, and C-fiber high-threshold mechanoreceptive (HTM) units that responded to noxious mechanical stimuli but not to heat; and (2) Aδ- and C-fiber units that responded to both noxious mechanical and heat stimuli, further subdivided into: (a) C-fiber units responsive to superficial mechanical and heat stimuli (C-polymodal nociceptors); (b) C-fiber units responsive to deep mechanical (likely dermal) and heat stimuli (C-mechano-heat units); and (c) Aδ-fiber mechano-heat units with either superficial or dermal receptive fields. All these subgroups of nociceptive neurons were clearly identifiable in both normal and CFA-treated animals. Neurons unresponsive to any of the aforementioned noxious or non-noxious stimuli were excluded from the study. At the conclusion of the experiments, animals were euthanized with an overdose of anesthetic. Neurons were included in the analysis only if their receptive fields were located within the inflamed area (the paw and leg, but not the hip) and if they had a resting membrane potential (Em) of at least −40 mV, an overshooting AP, and an AHP. Unlike in previous studies [8, 13], in which C-fiber neurons without AHP were included due to the limited number of subgroups, only neurons with AHP were considered in this study to allow for better comparison between subtypes. Neurons with cutaneous receptive fields over the hip (outside the inflamed area) were excluded from analysis. Based on dorsal root CVs, rat neurons were classified as C (≤0.8 m/s), Aδ (1.5–6.5 m/s), or Aα/β (>6.5 m/s). This classification was derived from compound APs recorded from L5 and L6 dorsal roots in normal rats of similar age and weight to CFA-treated rats, using identical experimental conditions [23], as previously described [8]. In guinea pigs, DRG neurons were similarly classified based on dorsal root CVs as C (<1.1 m/s), Aδ (1.1–4.2 m/s), or Aα/β (>4.2 m/s). These values were obtained from compound AP recordings in S2 dorsal roots of normal guinea pigs with comparable age and weight to CFA-treated animals [8]. The CVs in guinea pigs were lower than those in rats, likely due to a combination of factors: (1) younger animal age, (2) lower paraffin pool temperature, (3) inherent differences between dorsal root and peripheral nerve CVs, and (4) the inclusion of utilization time, as previously reported [8, 13].

### Statistical analysis

**Figure 2.**
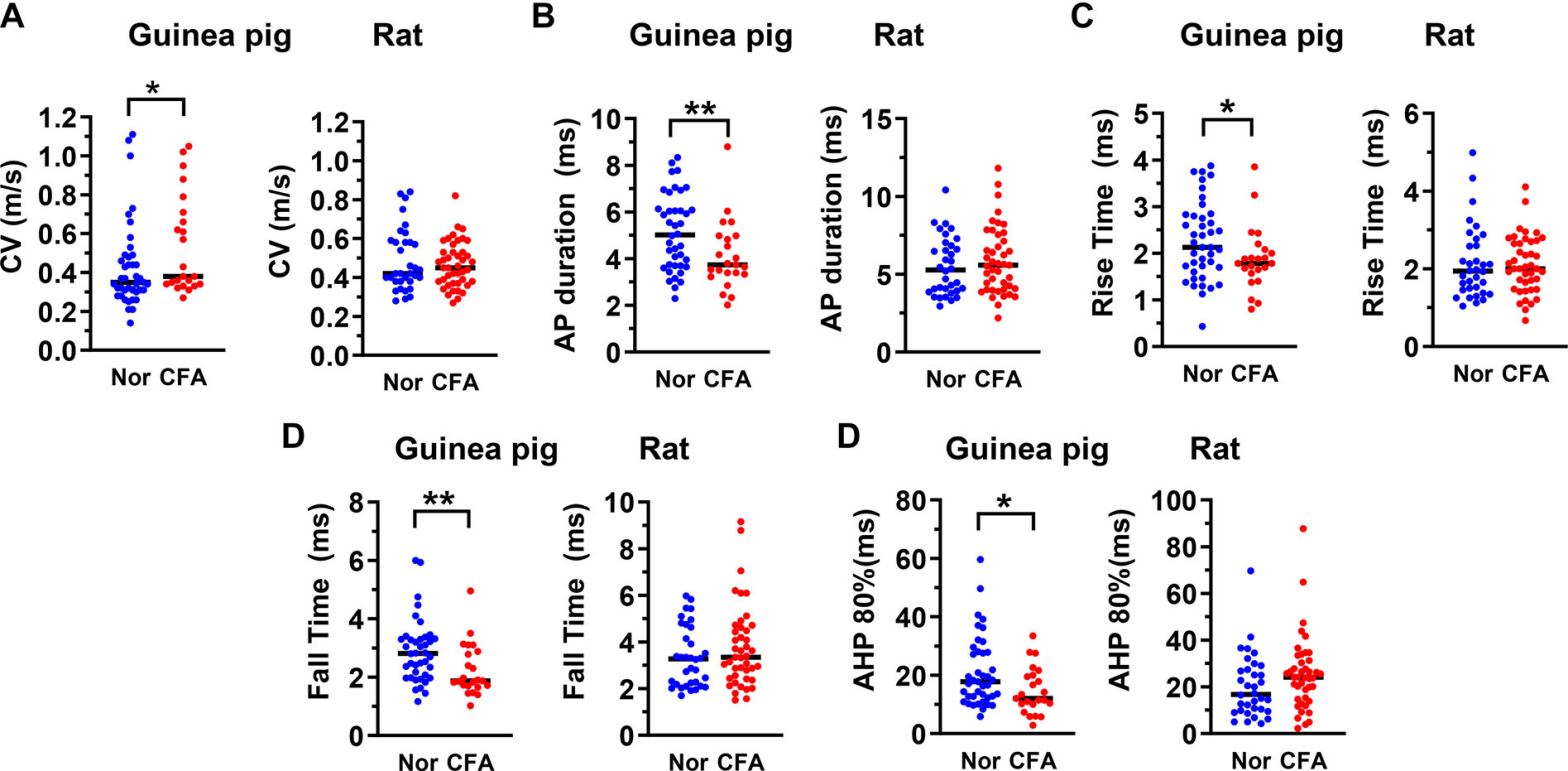
**Impact of CFA on CV and AP variables in C-fiber nociceptive neurons in the guinea pig and rat**. Scatterplots showing the effects of CFA treatment on CV (A) and AP variables that changed significantly in C-nociceptive neurons: AP duration at base (A), Rise time (C), Fall time (D), and AHP 80% (E). Each dot represents the value for one DRG neuron. Nor means untreated/normal animals, and CFA means CFA injection 4 days prior to the electrophysiological experiments. The median (horizontal line) is superimposed in each case, and the level of significance of any difference between normal animals (Nor) and CFA treated animals (CFA), is indicated by asterisks above the graphs (no asterisks indicate no significant differences). Note that the median values of the variables shown changed significantly in the guinea pig (left panel), but not in the rat (right panel). Comparisons between normal and CFA groups were made with the Mann–Whitney *U* test. The level of statistical significance is as follows: *P < 0.05; **P < 0.01. CFA: Complete Freund’s adjuvant; DRG: Dorsal root ganglion; AP: Action potential; AHP: Afterhyperpolarization; CV: Conduction velocity.

**Figure 3.**
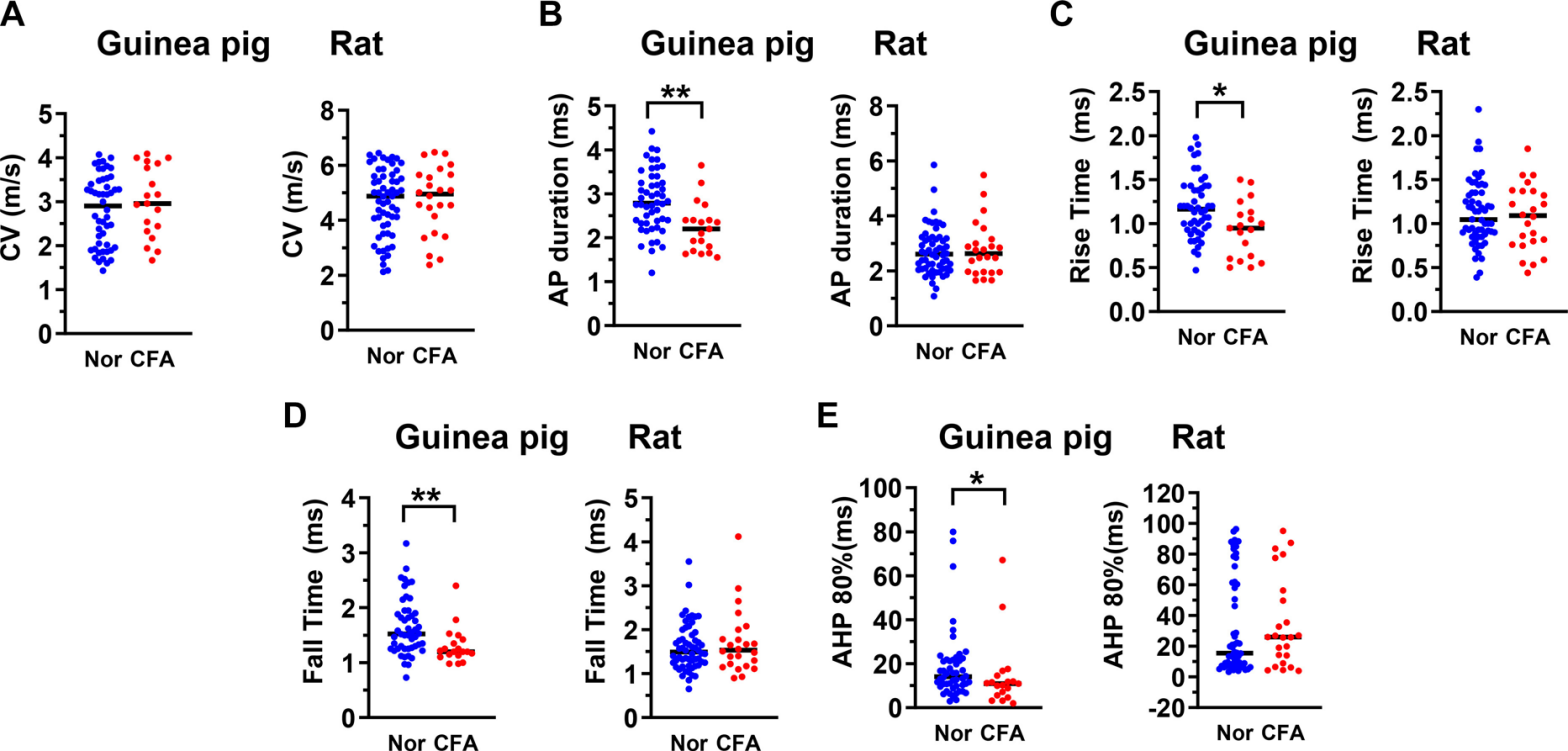
**Impact of CFA on CV and AP variables in δ-fiber nociceptive neurons in the guinea pig and rat**. Scatterplots showing the effects of CFA treatment on CV (A) and AP variables that changed significantly in δ-nociceptors namely AP duration at base (A), Rise time (C), Fall time (D), and AHP 80% (E). Like C-fiber nociceptors, the median values of these variables changed significantly in the guinea pig (left panel), but not in the rat (right panel). Each dot represents the value for one DRG neuron. Nor means untreated/normal animals, and CFA means CFA injection four days prior to the electrophysiological experiments. The median (horizontal line) is superimposed in each case, and the level of significance of any difference between normal animals (Nor) and CFA treated animals (CFA), is indicated by asterisks above the graphs (no asterisks indicate no significant differences). Note that the median values of the variables shown changed significantly in the guinea pig (left panel), but not in the rat (right panel). Comparisons between normal and CFA groups were made with the Mann–Whitney *U* test. The level of statistical significance is as follows: *P < 0.05; **P < 0.01. CFA: Complete Freund’s adjuvant; DRG: Dorsal root ganglion; AP: Action potential; AHP: Afterhyperpolarization; CV: Conduction velocity.

**Figure 4.**
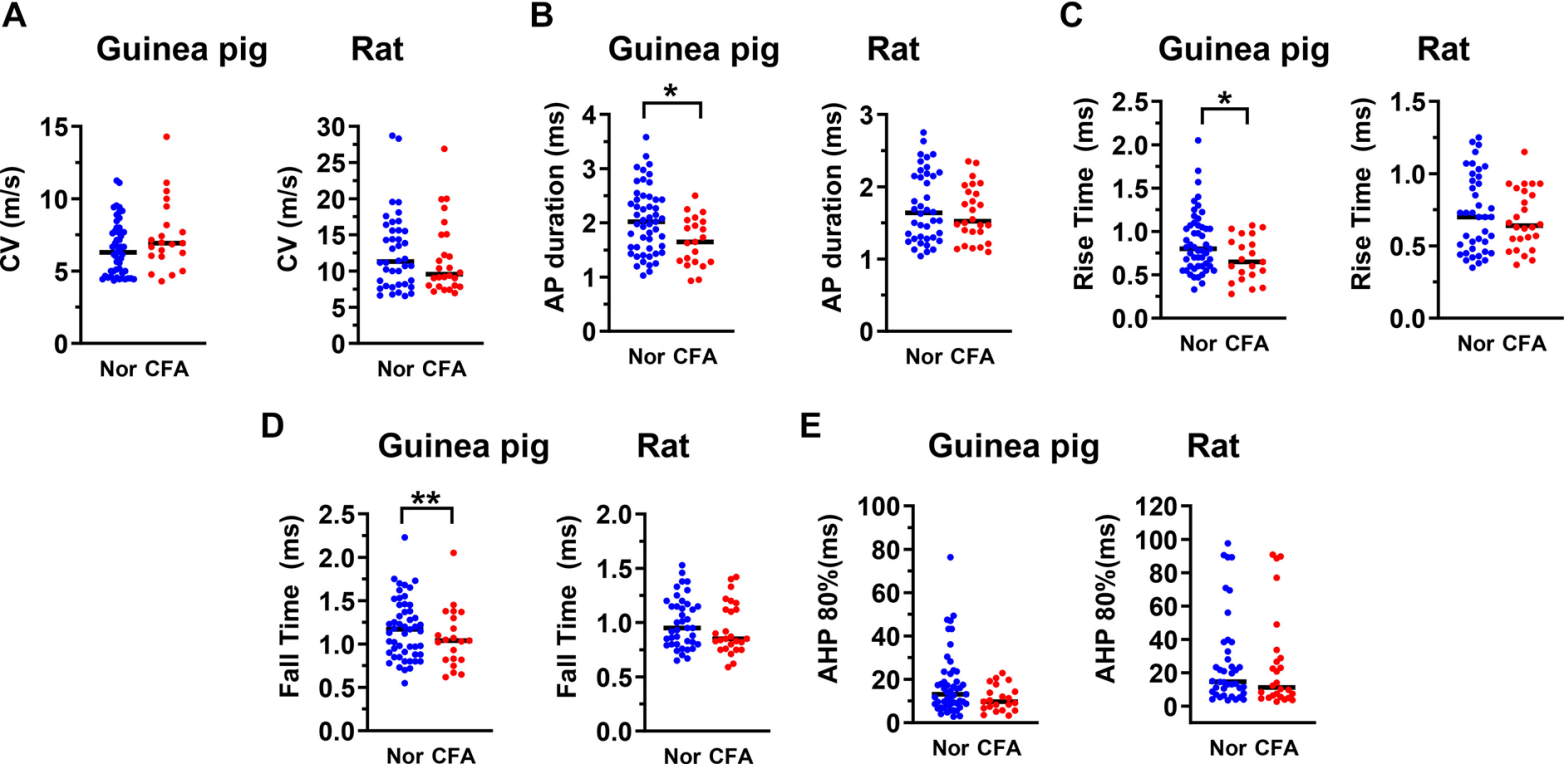
**Impact of CFA on CV and AP variables in α/β-fiber nociceptive neurons in the guinea pig and rat**. Scatterplots showing the effects of CFA treatment on CV (A) and AP variables that changed significantly in α/β-fiber nociceptive DRG neurons which are: AP duration at base (A), Rise time (C) and Fall time (D). Like C-fiber nociceptors, the median values of these variables changed significantly in the guinea pig (left panel), but not in the rat (right panel). Each dot represents the value for one DRG neuron. Nor means untreated/normal animals, and CFA means CFA injection four days prior to the electrophysiological experiments. The median (horizontal line) is superimposed in each case, and the level of significance of any difference between normal animals (Nor) and CFA treated animals (CFA), is indicated by asterisks above the graphs (no asterisks indicate no significant differences). Note that the median values of the variables shown changed significantly in the guinea pig (left panel), but not in the rat (right panel). Comparisons between normal and CFA groups were made with the Mann–Whitney *U* test. The level of statistical significance is as follows: *P < 0.05; **P < 0.01. CFA: Complete Freund’s adjuvant; DRG: Dorsal root ganglion; AP: Action potential; CV: Conduction velocity.

Most of the data in the control and experimental groups were not normally distributed and are therefore presented as medians. Comparisons were made using the nonparametric Mann–Whitney *U* test (Figures 2–4). All statistical analyses were performed using GraphPad Prism software, version 10 (GraphPad, San Diego, CA, USA). Significance levels are indicated above the graphs and in Table 1 as follows: *P < 0.05, **P < 0.01, ***P < 0.001. In Table 1, medians are reported, and variability is expressed as the 25th and 75th percentile values for each data set.

## Results

Intracellular recordings were obtained from a total of 225 nociceptive DRG neurons in rats and 205 in guinea pigs (see Table 1). These recordings were made in 23 normal (untreated) rats, 20 CFA-treated rats, 24 normal guinea pigs, and 21 CFA-treated guinea pigs. Among the rat DRG neurons, 77 were C-fiber nociceptors (43 from CFA-treated rats and 34 from normal rats), 82 were Aδ-fiber neurons (58 from normal rats and 24 from CFA-treated rats), and the remaining 66 were Aα/β-fiber nociceptors (40 from normal and 26 from CFA-treated rats). In guinea pigs, 64 units were C-fiber nociceptors (23 from CFA-treated animals and 41 from untreated animals), 69 were Aδ-fiber nociceptors (50 from normal and 19 from CFA-treated animals), and 72 were Aα/β-fiber nociceptors (51 from normal and 21 from CFA-treated animals).

### Hindlimb inflammation induces significant changes in electrophysiological variables in the guinea pig

Comparisons between variables recorded from nociceptive DRG neurons in normal/untreated guinea pigs (no CFA) and CFA-treated guinea pigs (four days post-CFA) are shown in Table 1 and Figures 2–4. As shown in Figure 2 and Table 1, C-fiber nociceptive neurons in CFA-treated animals had significantly lower median values than those in untreated animals for the following variables: AP duration (Figure 2B), AP RT (Figure 2C), AP FT (Figure 2D), and AHP at 80% recovery (AHP 80%; Figure 2E). In addition, C-fiber nociceptors in CFA-treated guinea pigs (but not in rats) showed a significant increase in CV (Figure 2A and Table 1) compared to untreated guinea pigs. In Aδ-fiber nociceptors (Figure 3), CFA treatment did not significantly affect CV (Figure 3A). However, as observed in C-fiber nociceptors, median values for AP duration (Figure 3B), AP RT (Figure 3C), AP FT (Figure 3D), and AHP 80% (Figure 3E) were significantly lower in CFA-treated animals compared to untreated controls (see also Table 1). Similarly, Aα/β-fiber nociceptors (Figure 4) displayed significantly reduced median values for AP duration (Figure 4B), AP RT (Figure 4C), and AP FT (Figure 4D) in CFA-treated animals compared to controls (see Table 1). However, the decrease in AHP 80% in this group was not statistically significant (Figure 4E).

**Table 2:** A summary of the changes in electrophysiological properties of nociceptive DRG neurons four days after CFA-induced hindlimb

**CV range**	**Animal** **group**	**N**	**CV** **m/s**	**Em** **(mV)**	**AP height** **(mV)**	**AP overshoot** **(mV)**	**AP duration** **at base (ms)**	**Rise time** **(ms)**	**Fall time** **(ms)**	**AHP depth** **(mV)**	**AHP duration 80% (ms)**
*C-fiber*	Rat normal Rat CFA	34 43	— —	— —	— —	— —	— —	— —	— —	— —	— —
	GP normal GP CFA	41 23	— ***↑***	— —	— —	— —	***↓↓***	***↓***	***↓↓***	— —	— ***↓***
*Aδ-fiber*	Rat normal Rat CFA	58 24	— —	— —	— —	— —	— —	— —	— —	— —	— —
	GP normal GP CFA	50 19	— —	— —	— —	— —	— ***↓↓***	— ***↓***	— ***↓↓***	— —	— ***↓***
*Aα/β-fiber*	Rat normal Rat CFA	40 26	— —	— —	— —	— —	— —	— —	— —	— —	— —
	GP normal GP CFA	51 21	— —	— —	— —	— —	— ***↓***	— ***↓***	— ***↓↓***	— —	— —

CFA: Complete Freund’s adjuvant; DRG: Dorsal root ganglion; AP: Action potential; AHP: Afterhyperpolarization.

### Hindlimb inflammation induces no significant changes in electrophysiological variables in the rat

Consistent with our hypothesis—and in sharp contrast to the guinea pig—there were no significant changes in any of the variables listed in Table 1 in CFA-treated rats (four days post-CFA) compared with untreated (no CFA) rats in any cardiovascular group. As shown in Table 1 and Figures 2–4, the median values of all measured variables in CFA-treated rats were not significantly different from those in untreated rats. Table 2 summarizes the changes in electrophysiological properties of nociceptive DRG neurons four days after CFA-induced hindlimb inflammation in both guinea pigs and rats. The observed differences in the electrophysiological properties of nociceptive DRG neurons following CFA-induced hindlimb inflammation suggest species-specific neuronal mechanisms underlying chronic inflammatory pain.

## Discussion

In this study, we used *in vivo* intracellular recordings to determine whether CFA-induced hindlimb inflammation produces electrophysiological changes in rat DRG nociceptors, as we previously observed in guinea pigs [8, 13]. We conducted a side-by-side comparison of the effects of CFA treatment on the electrophysiological membrane properties of nociceptive DRG neurons in guinea pigs and rats—two species with distinct genetic backgrounds. Consistent with our earlier findings in guinea pigs, we observed significant changes in several variables, including CV, AP, and AHP characteristics, four days after CFA-induced hindlimb inflammation. However, in line with our hypothesis, we found no significant changes in any of the variables measured in rat nociceptors. This suggests species-specific differences in the impact of tissue inflammation on the electrophysiological properties of nociceptive DRG neurons, and potentially, differences in the peripheral neuronal mechanisms underlying inflammatory pain. These observed differences may arise from species-specific variations in ion channel expression and/or function in the DRG. Genetic differences between the two species may lead to differential expression of receptors, ion channels, and signaling molecules involved in nociception. The inflammation-induced changes in AP and AHP properties in guinea pig DRG nociceptors are likely attributable to alterations in the expression and/or biophysical properties of various ion channels. These include the Na^+^ channel Nav1.8, which contributes to AP RT and overshoot in most nociceptive afferents [24], and Ca^2+^-dependent K^+^ channels, as well as delayed-rectifier K^+^ channels, which respectively mediate AHP and AP repolarization in sensory neurons [25]. Several studies have shown that Nav1.8 plays a critical role in inflammation-induced hyperexcitability of afferent sensory neurons and in inflammatory pain. For instance, models of inflamed hind paw have demonstrated upregulation of Nav1.8 expression and an associated increase in the slowly inactivating TTX-resistant current in DRG neurons [26], along with enhanced Nav1.8 immunoreactivity, particularly in unmyelinated axons [27]. Given that such inflammation-induced increases in Nav1.8 expression and TTX-R current occur in rat DRG neurons, our finding of no significant changes in AP and AHP variables in rats following CFA-induced inflammation was unexpected. The striking contrast in the effects of cutaneous inflammation on the electrophysiological properties of DRG neurons in guinea pigs vs rats may reflect differences in the expression and/or function of the aforementioned ion channels and others involved in AP and AHP regulation, as previously reported in CNS neurons. To our knowledge, this is the first study to report species differences in the electrophysiological response of DRG neurons to cutaneous inflammation. However, species-related differences in the electrophysiological properties of CNS neurons have been documented. For example, significant differences between neurons in the dorsal motor nucleus of the vagus (DMV) in rats and guinea pigs have been described [28]. These include: (1) larger amplitude and broader APs in guinea pig neurons, suggesting increased Ca^2+^ entry during APs; (2) longer AHP durations in guinea pig neurons, contributing to their slower repetitive firing; (3) the presence of two Ca^2+^-activated K^+^ currents (Gk_ca,1 and Gk_ca,2) in most guinea pig neurons, compared to only the apamin-sensitive Gk_ca,1 in rats [29]; (4) a larger inward rectifier current in guinea pig neurons than in rat neurons; and (5) a more prominent hyperpolarization-activated current (Ih) in guinea pig DMV neurons. Another *in vitro* electrophysiological study using whole-cell recordings from central amygdala neurons [30] found that most central medial and lateral neurons in guinea pigs exhibited an outward rectification current that delayed firing onset in response to depolarizing current pulses. In contrast, these so-called late-firing neurons were rare in the rat central nucleus.

It is noteworthy that several other differences between guinea pigs and rats have been previously reported, including marked differences in the cytochemical properties of their DRG neurons [31]. For instance, guinea pigs exhibit significantly higher levels of the neurotransmitter substance P (SP) in the DRG, dorsal roots, and dorsal spinal cord. Additionally, guinea pigs show a 100- to 500-fold higher affinity for CP-96,345100—a selective antagonist of SP’s preferred receptor, neurokinin-1—compared to rats [32]. SP, a pain-related neurotransmitter, is found in both the peripheral and central terminals of C-fiber nociceptors. It plays a crucial role in pain transmission and neurogenic inflammation, with its levels increasing in inflammatory states and being associated with heightened pain sensitivity (for reviews, see, e.g., [33, 34]). Species differences have also been reported in the expression of P2X5 receptors—ATP receptor subtypes involved in transmitting pain signals from the periphery to the spinal cord [35]. For example, an immunohistochemical study examining P2X5 receptor expression in DRGs across several mammalian species, including rats and guinea pigs, found that P2X5 receptor levels are higher in guinea pig DRGs than in those of rats [36]. As previously noted, rats and mice are more commonly used in biomedical research as animal models of pain (see e.g., [18]). This preference is due to the more extensive characterization of their genomic, proteomic, and metabolomic profiles, as well as better understanding of their system functions and behavior. Additionally, rats and mice are considered evolutionarily closer to humans than most other non-primate mammals. Rats are favored over guinea pigs in pain research for several scientific and practical reasons, including: Rats display clearer and more quantifiable pain-related behaviors (e.g., licking and guarding) than guinea pigs; guinea pigs require specialized care and are more susceptible to stress, which can confound pain studies; and a broader range of immunohistochemical and genetic tools are available for rats than for guinea pigs. However, we have previously used guinea pigs in our research [8, 13], as they are, in many respects, more similar to humans than rats, mice, or even chimpanzees. For example, aspects of their immune system [37] and fetal developmental timing [38] are more comparable to those of humans. Metabolically, guinea pigs are also closer to humans, possessing cholesteryl ester transfer protein, lipoprotein lipase, and lecithin–cholesterol acyltransferase [39, 40]. Notably, along with primates, guinea pigs are the only laboratory animals with a dietary requirement for vitamin C, whereas rats lack plasma cholesteryl ester transfer protein [40]. A significant difference has also been reported between rats and guinea pigs in the brain’s histamine system, particularly in the regional distribution of histamine H1 receptors [41]. Moreover, unlike rats, guinea pigs are “precocial”—born with their eyes open and with relatively advanced brain development. Indeed, guinea pig brains resemble human brains in several ways, including the structure of the Circle of Willis [42]. The primary strengths of the current study include: (a) electrophysiological recordings of DRG neurons *in vivo*, i.e., in their natural environment, which is dynamic and not fully replicable *in vitro*; (b) physiological identification of nociceptors via their receptive properties, which cannot be assessed *in vitro*; and (c) a large sample size. One limitation of the study is the exclusive use of female rats and the recording of electrophysiological data at a single time point after CFA treatment (day four post-CFA). Although sex differences are an important and emerging area in chronic pain research—and recent evidence suggests sex-based differences in the electrophysiological properties of human DRG neurons [43]—this issue is beyond the scope of the present study. We used female animals for practical reasons: we found laminectomy procedures to be easier in females, likely due to their softer bones.

## Conclusion

Our findings of significant changes in the electrophysiological properties of nociceptive DRG neurons in guinea pigs—but not rats—following hindlimb inflammation may stem from species-specific differences in ion channel expression and/or function. As previously suggested [8, 13], inflammation-induced decreases in AP and AHP durations—effectively shortening the absolute and relative refractory periods—are likely to increase the firing frequency of nociceptive nerve fibers, thereby contributing to pain hypersensitivity. In other words, these inflammation-induced changes may enhance the capacity of nociceptors to transmit signals to the CNS, promoting inflammatory pain hypersensitivity in guinea pigs. Our results highlight species-specific differences in the peripheral neuronal mechanisms underlying inflammatory pain. However, further comparative studies are essential to clarify these interspecies differences and similarities, which could inform the selection of appropriate animal models for chronic pain research and improve the translational relevance of preclinical findings.
